# Efficient and sustainable extraction of uranium from aquatic solution using biowaste-derived active carbon

**DOI:** 10.3389/fchem.2023.1327212

**Published:** 2023-12-20

**Authors:** Ashfaq Ahmad, Salah Ud-Din Khan, Rawaiz Khan, Nils Haneklaus

**Affiliations:** ^1^ Department of Chemistry, College of Science, King Saud University, Riyadh, Saudi Arabia; ^2^ Sustainable Energy Technologies Center, College of Engineering, King Saud University, Riyadh, Saudi Arabia; ^3^ Engineer Abdullah Bugshan Research Chair for Dental and Oral Rehabilitation, College of Dentistry, King Saud University, Riyadh, Saudi Arabia; ^4^ Td-Lab Sustainable Mineral Resources, Universität für Weiterbildung Krems Krems an der Donau, Austria

**Keywords:** biowaste, chemical activation, activated carbon, uranium, extraction

## Abstract

Efficient and cost-effective biosorbents derived from biowaste are highly demanding to handle various environmental challenges, and demonstrate the remarkable synergy between sustainability and innovation. In this study, the extraction of uranium U(VI) was investigated on biowaste activated carbon (BAC) obtained by chemical activation (phosphoric acid) using Albizia Lebbeck pods as biowaste. The biowaste powder (BP), biowaste charcoal (BC) and BAC were evaluated by thermogravimetric analysis (TGA), Fourier transform infrared spectroscopy (FTIR) and Brunauer-Emmett-Teller (BET) with nitrogen adsorption for thermal properties, chemical structures, porosity and surface area, respectively. The pH_PZC_ for acidic or basic nature of the surface and X-ray diffraction (XRD) analysis were performed for BAC. The morphological and elemental analysis were performed by scanning electron microscopy (SEM) and energy dispersive X-ray (EDX). The extraction of uranium U(VI) ions from aqueous solutions using BAC as sorbent was investigated by using different variables such as pH, contact time, initial uranium U(VI) concentration and BAC dose. The highest adsorption (90.60% was achieved at 0.5 g BAC dose, 2 h contact time, pH 6, 10 ppm initial U(VI) concentration and with 200 rpm shaking speeds. The production of this efficient adsorbent from biowaste could be a potential step forward in adsorption of uranium to meet the high demand of uranium for nuclear energy applications.

## Introduction

Nuclear energy is considered as an efficient and powerful source of clean and renewable energy which assumes a vital role in mitigating the global energy crises as well as reducing global emission (Leggett et al., 2016; Elhegazy and Kamal, 2022; Mathew, 2022). For the production of nuclear energy, uranium is the primary fuel used in the nuclear reactors. Therefore, continuous availability of uranium is critical to sustainable and long-term growth of nuclear energy. Generally, on the Earth uranium is found as ore. However, these reserves are limited, unequally distributed, and may exhaust in a hundred years. (Grancea and Hanly, 2018). The known on-land total uranium reserves around the world is only 7.6 million tonnes which is far less than the required amount to meet the demand of nuclear energy growth (Yuan et al., 2021). Therefore, exploring alternative sources of uranium is critical to ensure a sustainable supply of uranium and nuclear energy production.

Sea water contains about 4.5 billion tonnes of uranium which is considered as one of the most viable solutions to fulfil the growing demand of uranium (Abney et al., 2017; Li et al., 2023). However, the uranium ion concentration in seawater is approximately 3.3 ppb which is very low and seawater is highly saline (Wang et al., 2021). Therefore, efficient methods are required to extract uranium from seawater. Researchers have made numerous efforts to explore various approaches for extracting uranium including: ion exchange (Foster et al., 2020; Chen et al., 2022), chemical precipitation (Burns et al., 2016), solution extraction (Chen et al., 2018), membrane separation (Hsiue et al., 1989) and adsorption technique (Li et al., 2022; Yu et al., 2022). Adsorption-based techniques for uranium removal have been considered as one of the facile, economical, and efficient methods and received enormous attention in recent decades (Yuan et al., 2021; Zhu et al., 2023). Researchers have proven adsorption being an efficient method for the extraction of many other rare and precious metals including Pd(II) (Li et al., 2023b; Cheng et al., 2023), Ag (Shao et al., 2023) and gold (Li et al., 2023c). The adsorption method is mainly based on the properties of adsorbent material, which included the organic/inorganic adsorbents (Qin et al., 2020; Yang et al., 2022), biosorbents (Akl et al., 2021) nanostructure sorbents (Dat et al., 2018). Moreover, adsorbents based on carbon (Yang et al., 2019), silica (Bai et al., 2021) and natural minerals (Satpathy et al., 2022) have also been studied by researchers. However, the search for large scale cost effective adsorbents with adequate adsorption capacity to extract uranium from highly saline sea water with low concentration of uranium (Pu et al., 2022).

Carbon based materials are considered excellent adsorbent because of their non-toxicity and ease of functionalization (Islam et al., 2021). Among them activated carbon (AC) has emerged as a promising candidate among various adsorbent materials owing to its chemical versatility, high surface area and porosity. Despite the promising properties of AC as an absorbent, the recovery of uranium by using AC exist some challenges. For instance, the presence of various ions and elements compete with the uranium for adsorption sites on the AC and prevent them from entering the micropores by clogging which leads to reduction in the efficiency of the AC. Similarly, the long-term reusability of AC without losing its adsorption capacity is another concern (Hassan et al., 2007). The production of AC from non-renewable sources (including coal or petroleum-based substrates) is a matter of great concern regarding environmental impact and sustainability. Therefore, renewable and environment friendly sources are required to address these issues. Lignocellulosic (LC) is the most abundantly available source of biomass as precursor for the production of cheap, sustainable and efficient AC. Many studies have documented the development of AC from a range of LC sources including palm oil shells, coconut shell, cotton stalks, bagasse of sugar cane, olive seeds, woods, date pits and hazelnut shells etc. (Yavuz et al., 2010; Carrier et al., 2012; Foo and Hameed, 2012; Ahmad et al., 2021).

This study presents uranium adsorption by using AC derived from biowaste (Albizia Lebbeck Pods) to address the environmental concerns related to uranium content as well as challenges associated with the sustainable production of adsorbent production. Herein, we prepared AC from local biowaste by chemical activation using phosphoric acid. The effectiveness of the obtained BAC as sorbent in batch technique for removing U(VI) ions from an aqueous solution was evaluated. Different sorption parameters, including pH of solution, contact duration, dosage and initial concertation of U(VI) were examined for their effects. The production of this efficient and economical adsorbent from biowaste will potentially enable the adsorption of uranium to achieve the high demand of uranium for nuclear energy applications.

## Materials and methods

The chemicals employed in this investigation were reagent grade ortho phosphoric acid (H_3_PO_4_ 85%), NaOH 97.5%, HNO_3_, (Grade: Analytical, BDH Chemicals, England) and hydrochloric acid. For the production of U(VI) stock solution (1,000 mg/L), UO_2_(CH_3_COO)_2_.2H_2_O (BDH Ltd. England) was utilized. No additional purification was performed on any of the chemical reagents. The solutions were made with Milli Q (Distilled and deionized) water.

### Preparation of BC and BAC

Biowaste (Albizia Lebbeck Pods) was obtained from botanical garden of University (King Saud University, Saudi Arabia). The biowaste were dried, washed and cut it into small pieces. Afterward milled into a fine particles and sifted through 0.180 mm mesh to obtain uniform particles. Six-gram BP was impregnated with 60% concentration of phosphoric acid for 24 h at room temperature (T_R_). The extra phosphoric acid solution was removed from the sample using vacuum filtration and the material was subjected to complete drying at 110°C in an oven. After complete drying, the sample was carbonized at 400°C temperature for 3 h at 5°C min^−1^ ramp rate in a muffle furnace. The developed carbonized sample was allowed to cool at normal ambient temperature. Lastly, the resulting sample was crushed in an agate mortar and pestle into fine powders and thoroughly rinsed many times using distilled water. The produced BAC was lastly washed with NaOH (0.5 mol L^-1^) to attain a neutral pH state of effluent. Washed BAC was dried in an oven at 110°C. The BC was produced by placing 6 g BP without any chemical impregnation in a crucible and carbonized in a muffle furnace at a temperature 400°C for 3 h with heating rate of 5°C per minute.

### Characterization methods

The BET surface area and pore volumes of the specimens (BP, BC, and BAC) were assessed by porosity and surface area analyzer (Micromeritics, Gemini VII, 2,390, United States of America). Before analysis, the sample was degassed for 60 min (N_2_ flow) at 150°C to remove humidity and fumes. The thermal stability of the BP was investigated by thermal gravimetric analysis (Mettler Toledo AG, Analytical, Switzerland) in temperature range 25°C–1,000°C at 10°C/min heating ramp rate (nitrogen environment). FTIR (Spectrum 100, Perkin Elmer, United States of America) was used to investigate the chemical composition of the samples. By adjusting the pH of BAC to a zero charge the point of zero charge (pHpzc) was determined. Moreover, X–ray diffraction (XRD) was conducted using X-ray diffractometer (D2 Phaser, Bruker, Germany). The samples were also evaluated for surface morphology and elemental analysis using a SEM-EDX (JED-2200 Series, JEOL, Japan).

### Batch U(VI) extraction experiments

Adsorption experiments for U(VI) were performed using a batch extraction. A 500 mL stock solution of UO_2_(CH_3_COO)_2_.2H_2_O was prepared at an initial concentration of 50 ppm in deionized water. The adsorption tests were performed in batch to evaluate the adsorption efficacy of BAC for U(VI) extraction from water. For this purpose, 15 mL of the stock U(VI) solution was introduced to a series of capped Erlenmeyer flasks (50 mL). The appropriate amount of BAC adsorbent was added to the flasks and placed in an oscillation chamber at a controlled temperature. After shaking, the suspension was filtered using filter paper. An inductively coupled plasma mass spectrometer (ICP-MS) was employed to examine and assess the content of the filtrates. The equilibrium and adsorption capacity of BAC (qe mg) and removal efficiency (removal %) were calculated using Eqs (1) and (2).

$$q_{e}=\frac{\left(C_{o}\;-\;C_{e}\right)V}{W}$$ (1)

By using Eq. 2, the percentage of uranium removal was determined.

$$\text{Removal }\left(\%\right)=\frac{C_{o}\;-\;C_{e}}{C_{o}}\times 100$$ (2)

Where Co = adsorbate solution concentration, Ce = Concentration at equilibrium (mg/L) W = utilized adsorbent weight (g), and V = solution volume (L).

The potential of BAC for U(VI) adsorption was evaluated against various operating variable (adsorbent dosage (0.1–0.8 g), U(VI) concentration (10–100 ppm), pH (2–9) and contact time (30–240 min)). The desired pH level of the solution was attained by introducing various amounts of HCl (0.1 M) and NaOH (0.1 M).

## Results and discussion

### Thermogravimetric analysis (TGA) of biowaste

**FIGURE 1:**
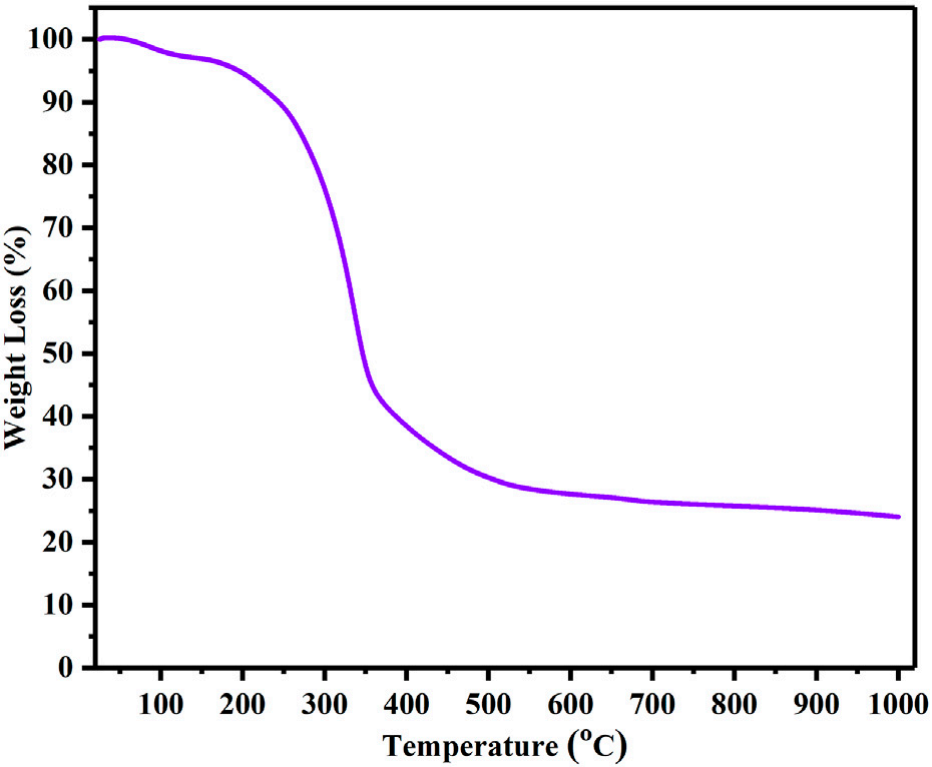
Thermogravimetry plot of Biowaste (BP).

The thermal properties of biowaste (BP) were evaluated by thermogravimetric analysis (TGA). The BP material was heated from 25°C to 1,000 °C in an inert N_2_ atmosphere at a ramp rate of 10 °C per minute. The obtained TGA thermogram was evaluated to obtain an optimized carbonization temperature. Figure 1 depict the TGA thermogram for BP, which demonstrates the tripartite weight loss of BP. In the first stage, the weight loss occurred between 25°C and 194°C, which accounted for weight loss of about 3.50%. This weight loss could be associated to the evaporation of moisture content and traces of volatiles. The second stage of weight loss started at 195°C and ended at 355°C, resulting in a significant weight loss of about 56.38%. This high weight loss could be associated to the structural break down of lignocellulose in BP. Typically, this break down process starts at a temperature above 210°C and leads to the conversion of cellulose, lignin and hemicellulose into gases and tars. In addition, the development of carbon material begins at this stage. The third and final stage of weight loss corresponds to lignin degradation, which begins at temperatures above 350°C–1,000°C. At this stage, the weight loss gradually decreases, with a weight loss of 20.25% and a plateau is reached. The TGA thermogram reveals that the conversion of biomass to carbon essentially occurs at 400°C.

### Textural analysis

The textural properties of the samples (BP, BC, and BAC) are illustrated in Table 1. These properties encompass BET surface areas (S_BET_), micropores, mesopores, surface areas, total pore volumes, and volumes of mesopores and micropores. The analysis was carried out by N_2_ adsorption-desorption isotherm (ADI) method. The S_BET_ 3.78 m^2^g^-1^, V_t_ 0.0012 cm^3^g^-1^, V_Meso._ 0.0012 cm^3^g^-1^ and S_BET_ 8.33 m^2^g^-1^, V_t_ 0.0031 cm^3^g^-1^, V_Meso._ 0.0031 cm^3^g^-1^ were obtained for BP and BC respectively. There is no micropore surface area and micropore volume in both samples (BP and BC). The S_BET_ and V_t_ of the H_3_PO_4_ activated BAC enhanced significantly. The surface areas (S_BET_ 1405.95 m^2^g^-1^, S_Mic._ 128.13 m^2^g^-1^ S_Meso._ 1277.82 m^2^g^-1^) and pore volumes (Vt 1.276 cm^3^g^-1^, V_Mic._ 0.048 cm^3^g^-1^, V_Meso._ 1.228 cm^3^g^-1^) were achieved for BAC synthesized by 60% H_3_PO_4_ concentration, 24 h soaking time, carbonization at 400°C and carbonization time 3 h. The mesopore volumes (V_Meso_) were calculated according to Eq. 3. The relatively small surface area and limited porosity of BP and BC can be ascribed to the densely packed structure of biowaste. The rise in BET surface area and porosity indicates that the initially dense structure of biowaste underwent a transformation, resulting in a significantly more porous structure. This disparity in surface area and porosity between the treated and untreated biomass was a direct outcome of the chemical treatment. During this process, the H_3_PO_4_ penetrated deep into microstructure of biowaste, facilitating the creation of new pores and, subsequently cause expansion in surface area (Ahmad et al., 2021).

$$V_{\text{Meso}.}=V_{\text{Total}}\;–\;V_{\text{Mic}.}$$ (3)

**TABLE 1:** The surface areas and pore volumes of biowaste.

No.	Materials	S_BET_ (m^2^/g)	t-Plot S_Mic._ (m^2^/g)	t-Plot S_Meso._ (m^2^/g)	V_P.Tot._ (cm^3^g^-1^)	t-Plot V_Mic._ (cm^3^g^-1^)	t-Plot V_Meso._ (cm^3^g^-1^)
1	BP	3.78	0.00	3.78	0.0012	0.00	0.0012
2	BC	8.33	0.00	8.33	0.0031	0.00	0.0031
3	BAC	1405.95	128.13	1277.82	1.276	0.048	1.228

Note: S_BET_: BET, surface area, S_Mic._and S_Meso_.: surface area corresponds to micro and meso pores, V_P. Tot._: Pores volume in total, V_Mic._ and V_Meso_: volume corresponds to micro and mesopore.

Where V_Total_ is the total pore volume, V_Meso._ is the mesopore volume, V_Mic._ = micropore volume.

### Nitrogen ADI

**FIGURE 2:**
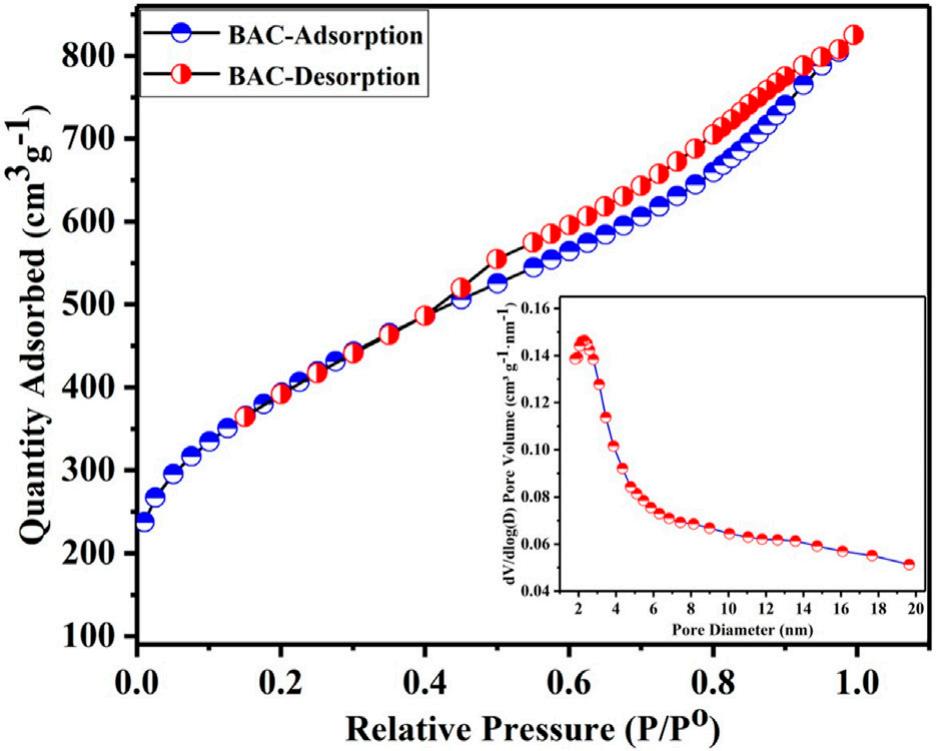
Nitrogen adsorption-desorption isotherm of BAC.

The N_2_ ADI is a useful tool for analyzing powder materials. Figure 2 depicts the N_2_ ADI at 77 K, which provides insights into the porosity of the produced BAC, along with the corresponding BJH pore size distribution (PSD) curve (shown in Figure 2 as an inset). The amount of N_2_ adsorption desorption of BAC is calculated in relation to the relative pressure (P/P^o^) of nitrogen. At low relative pressures (P/P^o^) less than 0.1, the isotherms displayed a small adsorption of nitrogen, indicating the existence of a limited number of micropores in BAC which is the characteristics of a type I isotherm. But, at above 0.4 P/P^o^, there was a substantial increase in N_2_ adsorption and hysteresis loop was observed during nitrogen adsorption and desorption processes. The N_2_ uptake quantity of BAC continued to rise with the relative pressure until it reached 0.6. The pronounced upturns (knees that are extensively opened) and the small hysteresis loops observed at relative pressures between 0.5 and 1.0 specified the existence of a significant quantity of mesopores in the BAC (Shrestha et al., 2019). The isotherm demonstrated that the BAC comprises of a substantial number of mesopores, in addition to some micropores. The ADI of the BAC exhibited characteristics of both type I and type IV isotherm as per IUPAC categorization. A type IV isotherm with an H4 hysteresis loop was observed at intermediate and high P/P^o^. This indicated a process involving mono and multilayer uptake through capillary condensation in fine, slit-type pores (Haggag et al., 2021). This suggests that the BAC has homogenous mesopores with narrow PSD of 2.3 nm, as shown in the insets in Figure 2.

### FTIR analysis

**FIGURE 3:**
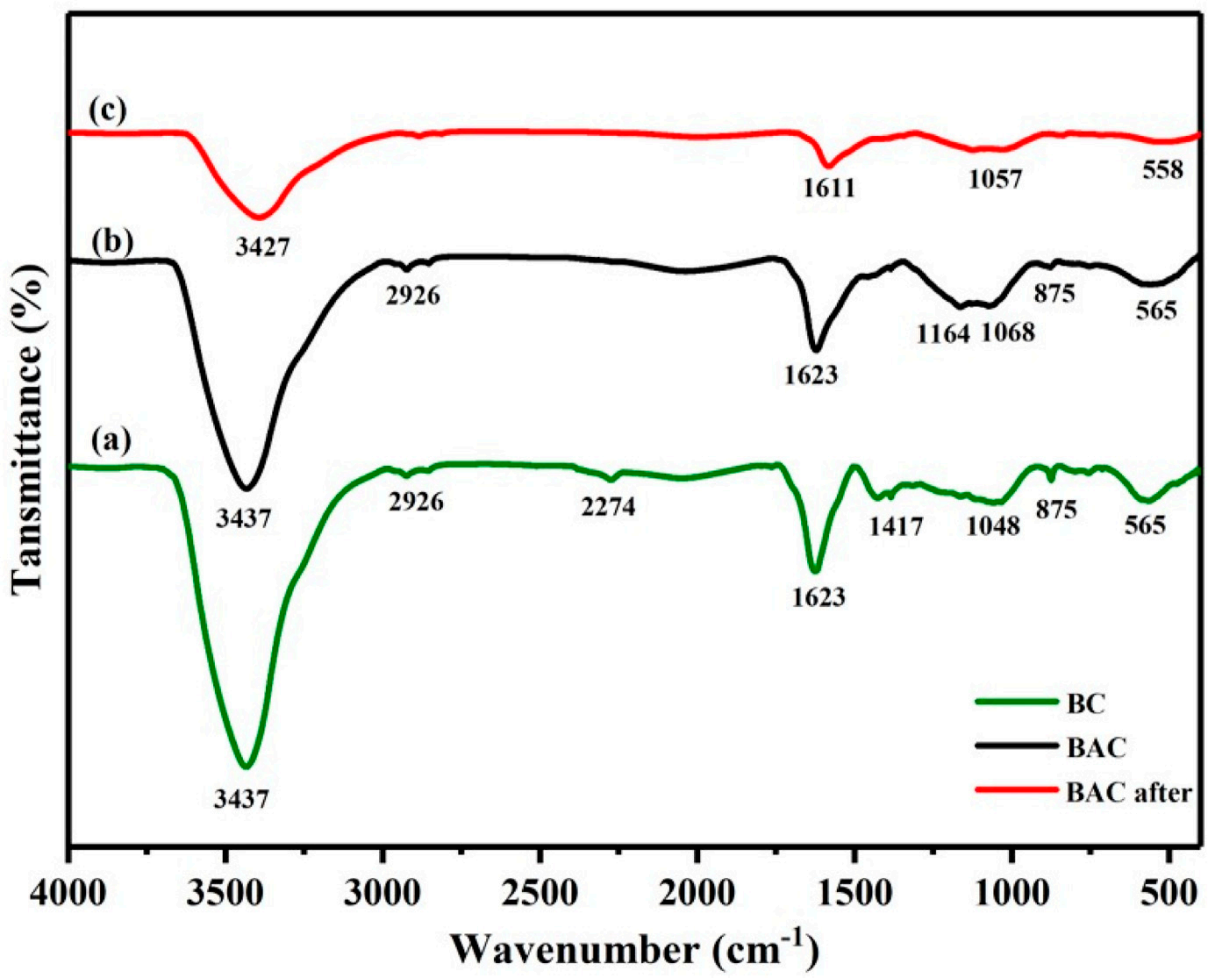
FTIR spectra of BC and BAC (before and after adsorption).

The FTIR spectra were acquired within the frequency range of 4,000 to 400 cm^−1^. The FTIR spectra displayed in Figures 3A–C compare the characteristics of BC and BAC. In Figure 3, both spectra (a,b) exhibit a prominent and broad absorption band at approximately 3,437 cm^−1^. This band’s location signifies the stretching and vibration of OH found in phenol, alcohol, and carboxyl compounds. Additionally, this −OH band can be associated with the absorbed water content in the raw BP specimen, which was corroborated by TGA analysis. The appearance peaks at 2,926 cm^−1^ is attributed to symmetric and asymmetric −CH vibrations in alkyl like methyl and methylene. The small band at 2,274 cm^−1^ corresponds to the NCO stretching and vibration on BC spectra (Li et al., 2023). Meanwhile, the broad bands in both spectra at approximately 1,623 cm^−1^ arises from conjugated alkene C═C stretching, and the small band at 1,417 cm^−1^ on BC spectra corresponds to the–CH bending. The band at around 1,164, 1,086 and 1,048 cm^−1^ in BC and BAC spectra could be associated to the vibration of the–CO bond, common in phenols, alcohols, esters, acids and ethers. The small intensity band at 875 cm^−1^ on both spectra belong to–CH out-of-plane bending vibration in aromatic compounds. In addition, the BAC and BC have peaks at 565 cm^−1^ region that corresponds to double six-member rings or monosubstituted aromatic rings (Lakhera et al., 2015; Gautam et al., 2021). These results align with previous research findings (Mohtashami et al., 2018; Ahmad et al., 2021). The spectrum depicted for BAC in Figure 3B exhibits a comparable set of peaks as BC. However, these peaks appear at lower absorption levels since a significant portion of the functional groups has been reduced through chemical activation. An analysis of the FTIR spectrum unmistakably reveals the presence of oxygen in various group forms, including, phenol, alcohol, ether and carbonyl. The FTIR spectra in Figure 3B, C illustrate the adsorbent (BAC) before and after the adsorption of U(VI), respectively. The various peaks corresponding to functional groups detailed in curve Figure 3B have already been explained. The BAC after adsorption of U(VI) did not show any new bands in the FTIR spectrum (Figure 3C). However, a notable reduction in transmittance and a slight peaks shift were observed. It can be deduced that the structure of the BAC remains unchanged after the sorption of U(VI). The absorption peaks wavenumber at 3,437, 1,623, 1,068 and 565 were slightly shifted to 3,427, 1,611, 1,057, and 558 respectively, suggesting that the substitution of H^+^ by UO_2_^2+^ results in a reduction in vibration intensity.

### X-ray diffraction (XRD) measurement

**FIGURE 4:**
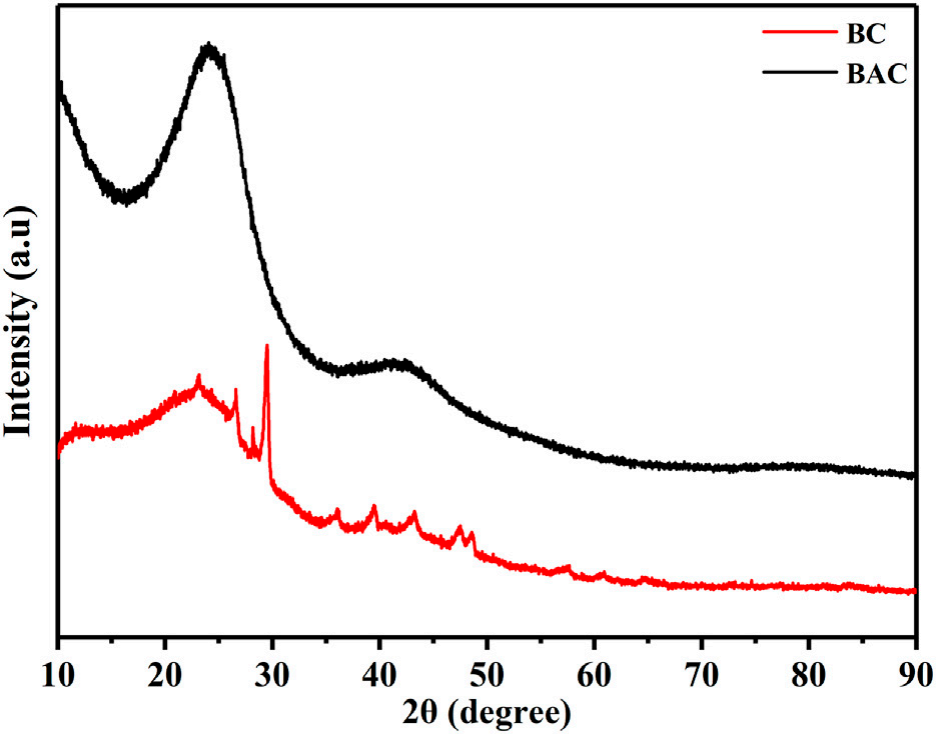
XRD patterns of BAC and BC.

XRD measurements of BC and BAC were performed within in the 2θ region spanning from 10° to 90° to assess the complete diffraction pattern of the samples as shown in Figure 4. The XRD patterns did not display prominent peaks within any range, particularly those associated with crystalline phases. This suggests that the samples did not exhibit any detectable mineral peaks. Pure activated carbon is noted for its broad peaks without distinct sharp spikes, indicating its amorphous nature, which can be advantageous for creating precise adsorbents (Tongpoothorn et al., 2011). The appearance of two broad bands at the 2θ around 24° and 43.2° in the case of BAC correspond to the graphitic carbon (002) planes and (100) planes, respectively (JCPDS No. 75–1,621), shows that carbon layer planes are aligned (Buasri et al., 2012). The BAC samples displayed broad peaks and the absence of sharp ones, signifying a primarily amorphous structure. This characteristic is beneficial for creating clearly defined porous materials. The pattern of BC also shows two peaks at the 2θ around 23° and 42° regions. Nonetheless, there were a few minor peaks discernible, which might be attributed to the existence of minor contaminants or water contents in the specimen (Shrestha et al., 2019).

### pH_PZC_ analysis

**FIGURE 5:**
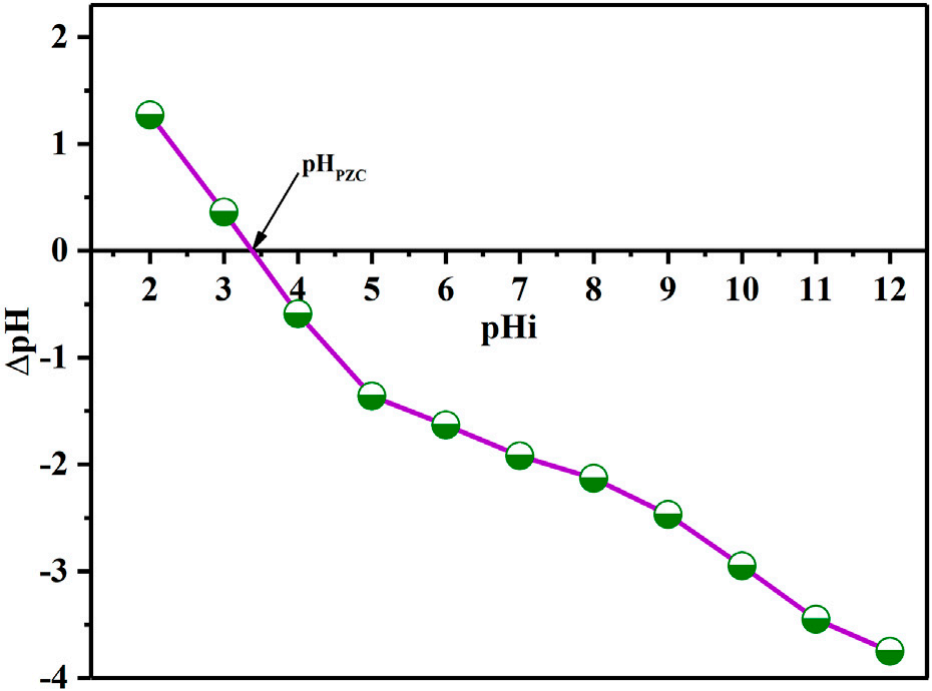
pHpzc of BAC.

The pH_PZC_ is a significant parameter that determines the pH sensibility range and enables for the prediction of surface active and adsorption capabilities (Bohli et al., 2015). The batch equilibrium technique was adopted to calculate pH_PZC_ (Doke and Khan, 2017). In various flasks, portions of 25 mL of KNO_3_ (0.01 M) solution was introduced into each flask. The pH was controlled within the target range of 2–12 by the addition of either NaOH or HCl solution (0.1 M each) followed by addition of 0.1 g of BAC. The solutions were transferred to oscillation chamber and kept 48 h at T_R_ under shaking to equilibrate the system. After filtering the dispersions, the pH of the resultant solutions (pH_f_) was then identified. Finally, the ∆pH = (pH_f_ – pH_i_) curve as a function of initial pH (pH_i_) was created Figure 5. The pH at pH_PZC_ was obtained using the curve’s point of intersection. The pHpzc value of BAC was determined to be 3.36.

### SEM and EDX analysis

**FIGURE 6:**
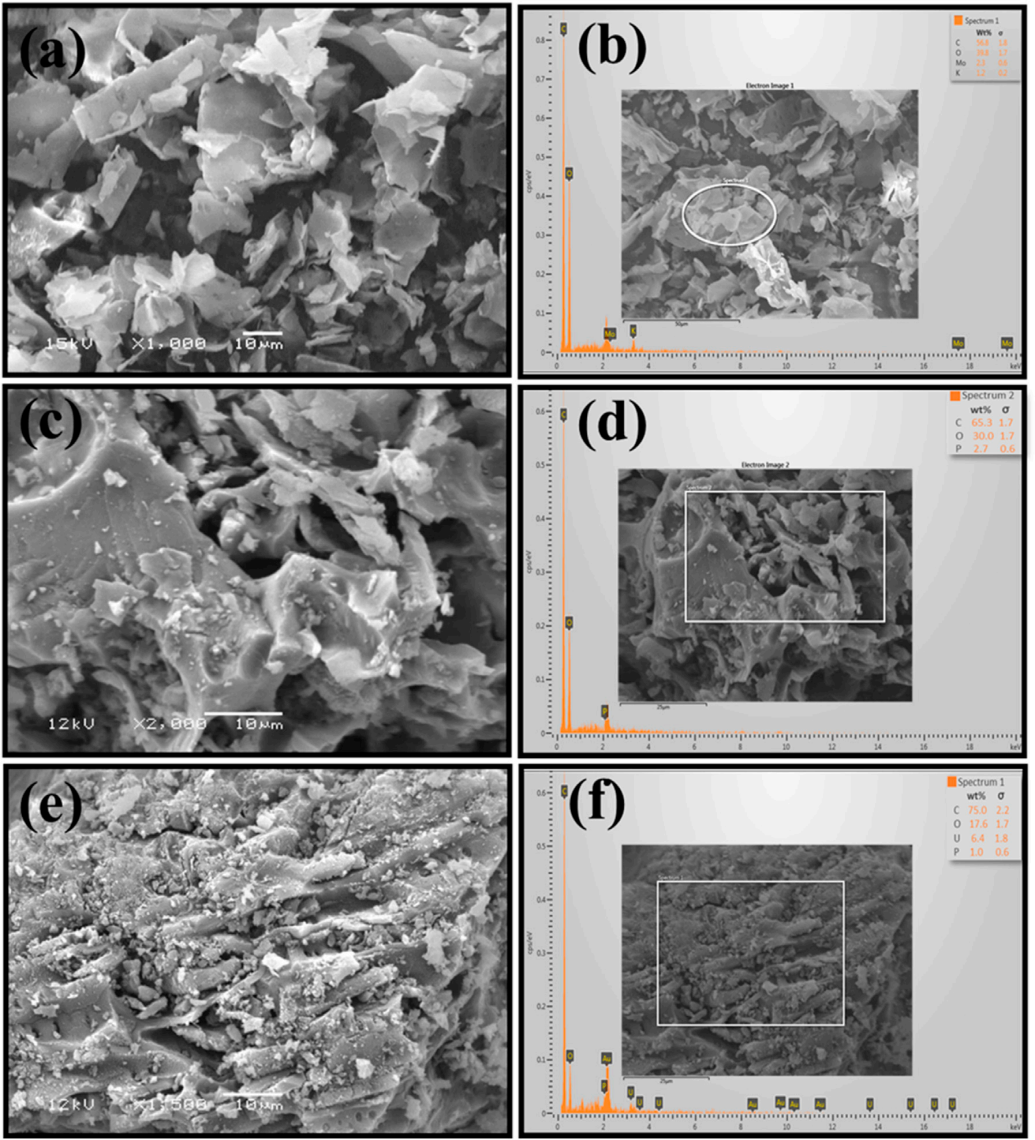
SEM micrographs and EDX analysis of BP and BC **(A)** BP SEM micrograph, **(B)** EDX of BP, **(C)** BAC SEM micrograph, before adsorption **(D)** EDX of BAC before adsorption **(E)** BAC SEM micrograph, after adsorption **(F)** EDX of BAC after adsorption.

Particle morphology is strongly influenced by surface properties. To assess the structure and elemental analysis of BP and BAC, SEM and EDX evaluations were conducted. Figures 6A–F shows the SEM micrographs and the EDX results of BP and BAC (before and after adsorption). From SEM micrographs, it is evident that the pristine biomass BP particles have a curved, sheet-like geometry with smooth surface morphology. The surface of the particles was relatively solid and did not exhibit pores or fractures. On the other hand, the BAC micrographs revealed coarse and fine particles with porous surface structure, possibly due to evaporation of volatiles during carbonization. Moreover, the BAC exhibited smaller particles with porous, and irregularly layered structure, which may have resulted in increased surface area. These pores on the surface of BAC may have developed as a result of H_3_PO_4_, dehydration and reaction with oxygen to produce phosphoric anhydride (P_2_O_5_) (Gao et al., 2021). The sublimation of solid P_2_O_5_ to gaseous form and its release at 360 °C from the surface of the BAC results in pores formation (Sarkar et al., 2015). Moreover, during washing of BAC, micro and mesopores are created as a result of P_2_O_5_ hydrolyses and removal, generating free spaces which accounted for enhance surface area (Saleh and Danmaliki, 2016; Xie et al., 2018).

The elemental composition of BP and BAC (before and after adsorption) were evaluated by EDX analysis. The obtained results are depicted in Figure 6B, D, F. It can be inferred from the results that carbon (C) and oxygen (O) remained the predominant elements in both BP and BAC. This due to the fact that BP as precursor materials employed in this study to obtain BP and BAC has somewhat similar physicochemical characteristics. The amount of C and O in BP was measured as 56.8% and 39.8% respectively (Figure 6B). However, the amount of C (65.3%) in BAC was enhanced with reduction in O (30.0%) content as a result of chemical activation with H_3_PO_4_ (Figure 6D). The increment in the C content may be associated with the complete removal of organic constituents while the reduction in O content may be associated with the removal of moisture content from the biowaste powder and formation of C–C chain in BAC. The small amount of Phosphorus (P) in BAC obtained in EDX analysis is due to use of H_3_PO_4_ as an activating agent. The EDX analysis of BAC after adsorption confirmed the uranium adsorption onto the BAC (Figure 6F).

### Effect of pH on adsorption

**FIGURE 7:**
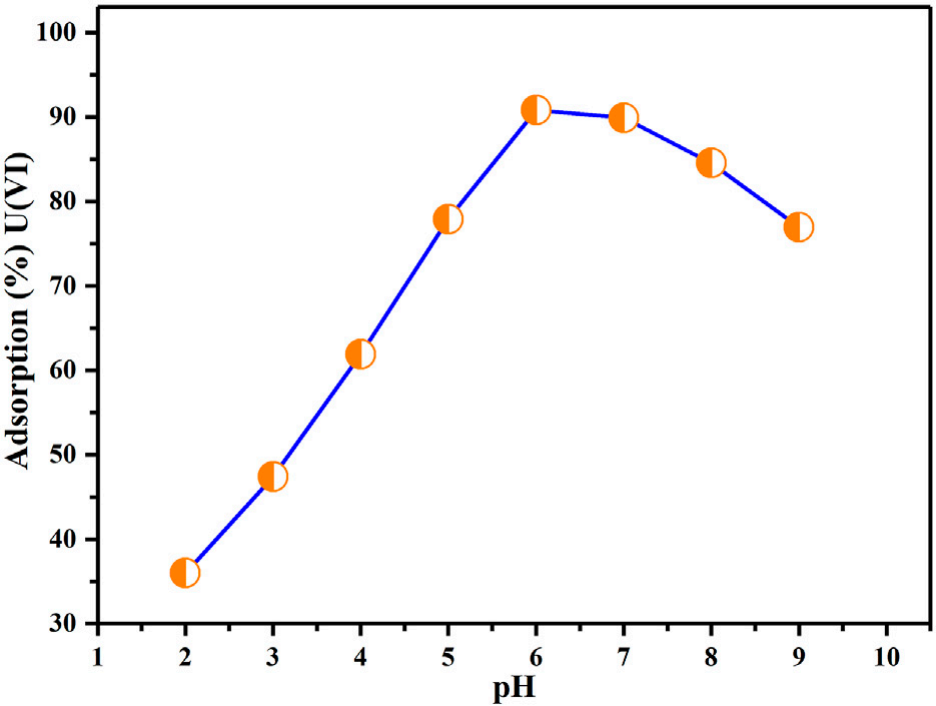
Influence of pH on extraction of U(VI) (Parameters: BAC dosage: 0.5 g, U(VI) initial concentration: 50 ppm, agitation speed: 200 rpm, contact duration: 2 h, at T_R_).

Figure 7 illustrates how different pH levels of the solutions influence the uptake of U(VI) by BAC. Figure 7 implies that the U(VI) extraction onto BAC significantly increases as the pH increases from 2 to 6. The maximum extraction efficiency for U(VI) ions was achieved at pH 6. Nonetheless, there is a subsequent decrease in extraction as the pH continues to rise from 6 to 9. The limited extraction of U(VI) at lower pH (less than 3.36) can be attributed to the high concentration and mobility of H^+^ ions, leading to competition for available adsorption sites with U(VI) ions., impeding the uptake of U(VI) ions by the BAC. Protonated adsorption places were incapable to bind U(VI) ions owing to electrostatic repulsion among uranium ions (+charged) and positively charged localities (Şen et al., 2015). As the pH enhanced, fewer hydrogen ions H^+^ were present in the solution, resulted in a higher number of negatively charged sites available. This helped the sorption of more uranium ions through electrostatic attraction forces. A decline in the extraction at higher pH levels is attributed to the creation of soluble hydroxy compounds. This behavior can also be elucidated by the charge properties of U(VI) and the adsorbent surface. At pH value around 3.4 for BAC, U(VI) is present as species with positive charge, while the surface of the adsorbent BAC is negatively charged. This results in an electrostatic force of attraction among the oppositely charged BAC surface and U(VI) ions, facilitating their adsorption onto the surface. When the pH exceeds 6.0, the dominant U(VI) are multi-nuclear carbonate and hydroxide complexes, leading to a declined extraction ability for U(VI) (Su et al., 2018). Consequently, all subsequent adsorption experiments aimed at achieving effective adsorption were conducted at a pH of 6.

### Impact of contact time

**FIGURE 8:**
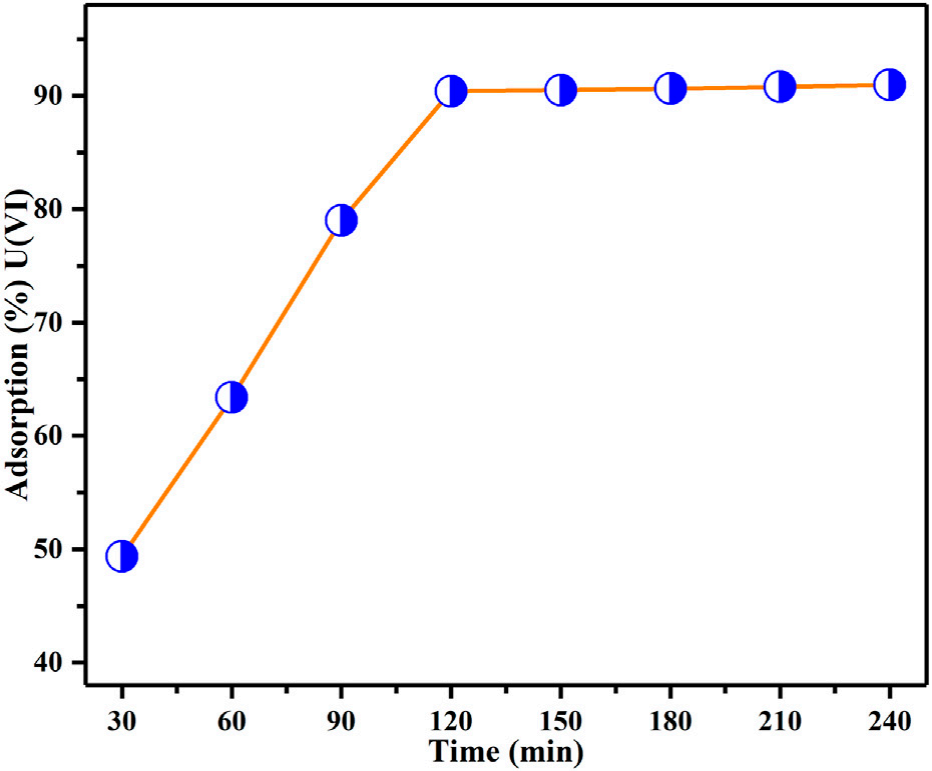
Impact of contact time on the extraction of U(VI) investigated under parameters: BAC dosage of 0.5 g, initial U(VI) content of 50 ppm, and a specified pH of the solution: 6, agitation speed: 200 rpm at T_R_).

Figure 8 illustrates the effect of different contact durations, spanning from 30 min to 240 min, on the adsorption of U(VI) by BAC. This parameter was investigated to determine the necessary contact time for achieving equilibrium between BAC and U(VI) ions within the solution. All other parameters (BAC dosage, initial concentration of U(VI), and pH) were maintained at constant levels. Figure 8 illustrates that the quantity of U(VI) removal increases over time until it reaches a certain level where no further extraction of U(VI) from the solution occurs. At this point, there is a dynamic equilibrium between U(VI) ions being subjected to adsorption by the adsorbent and those desorbed from it. The concentration of U(VI) ions captured at this equilibrium time underscores the high adsorption capacity of the BAC material (Ahmad et al., 2021). The findings indicate that U(VI) ion adsorption intensifies over time, reaching its peak around 120 min, after which it stabilizes for the remainder of the experiment. This suggests that initially, there is a rapid binding of the metal ions to the adsorbent material, which gradually drops and remains nearly constant after 120 min. As soon as the adsorbent material is introduced, the active sites are involved in binding the metal ions through complexation. However, further adsorption of metal ions occurs at a slower rate due to the limited number of active locations (Tchounwou et al., 2012).

### BAC dosage effect

**FIGURE 9:**
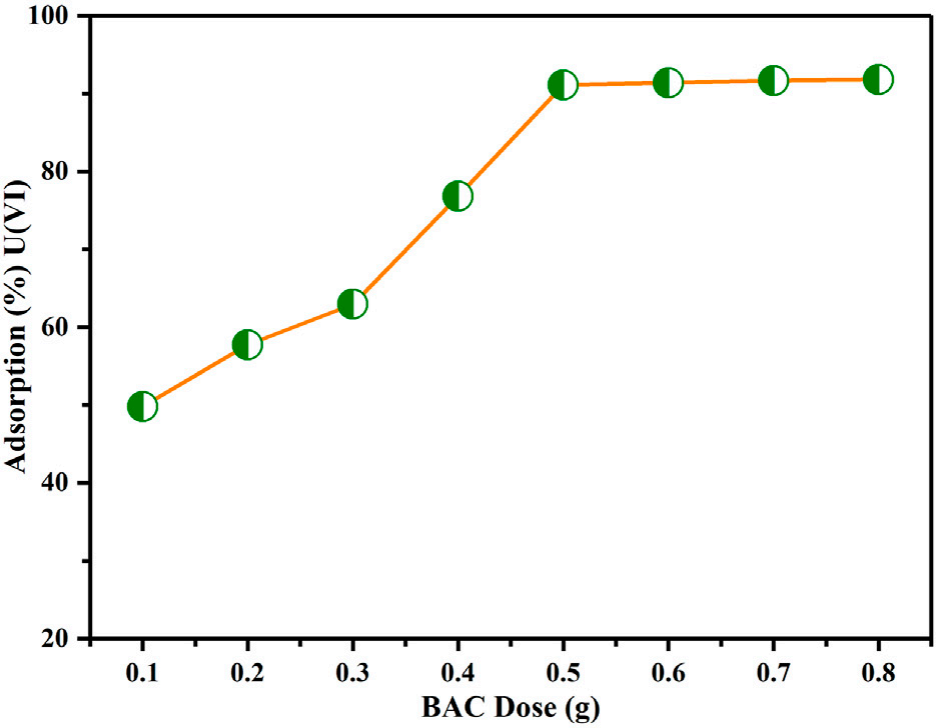
Influence of BAC dosage on extraction of U(VI) (Conditions: Contact time: 120 min, U(VI) initial concentration: 50 ppm, pH of solution: 6, agitation speed: 200 rpm at T_R_).

Figure 9 illustrates the impact of varying BAC dosage on the sorption of U(VI) ions. It is evident that the removal of U(VI) ion enhanced significantly, ranging from 50% to 90%, as the dosage is increased from 0.1 to 0.8 g. These results show that initially, there is a rapid extraction of U(VI) ions as the BAC dosage is increased. This is because, the increase in dosage produces more active sites for adsorption on surface for U(VI) ion binding (Saeidi et al., 2015). Furthermore, it is noticeable that there is no further rise in uranium ion adsorption when the BAC amount is increased beyond 0.5 g, possibly because of the overlap of active adsorption locations at elevated BAC doses. This overlapping result in reduction in the available active surface required for effective extraction (Khademi et al., 2015). Therefore, a dosage of 0.5 g for BAC sorbent was determined to be optimal for subsequent experiments.

### Initial U(VI) ion concentration effect

**FIGURE 10:**
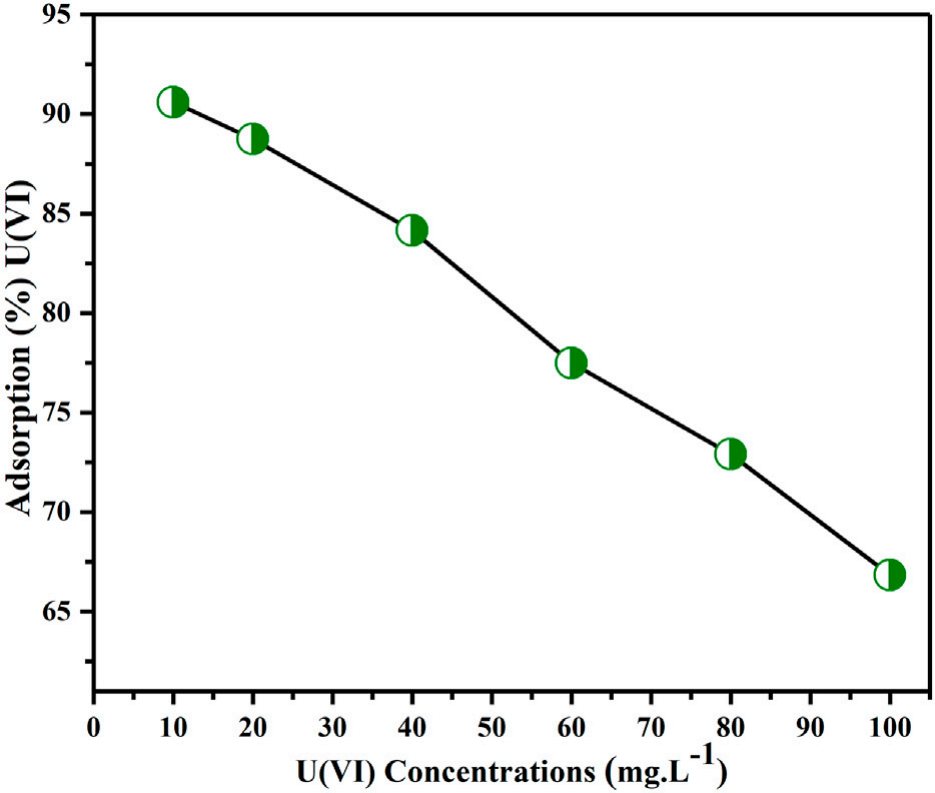
U(VI) concentrations impact on extraction of (Parameters: BAC dosage: 0.5 g, pH of solution: 6, contact time: 2 h, agitation rpm: 200 at T_R_).

Figure 10 depicts how the extraction effectiveness of U(VI) ions changed with concentration ranging from 10–100 mg L^-1^. The figure shows that, as earlier reported, when the concentration of uranium ion was increased from 10 to 100 ppm, its removal effectiveness declined from 90.6% to 66.83% (Wang et al., 2014; Tamjidi and Esmaeili, 2019). This occurs because, with low content of metal ions in the aqueous media, the adsorbent surface has a high proportion of active sorption sites in relation to the total ions. Consequently, all metal ions can form connections with the adsorbent and can be removed from the solution, thereby enhancing the results of the extraction. Conversely, when the concentration of metal ions is higher, there is a lower proportion of active sites available on the sorbent surface compared to the total number of metal ions in the aqueous solution. As a result, not all metal ions can engage with the adsorbent and be eliminated from the solution, leading to a decrease in the effectiveness of extraction (Wang et al., 2014). Consequently, a content of 10 mg L^-1^ was considered to be the ideal concentration for removing U(VI) from aqueous solutions using the BAC.

### Recycling and reusability of the BAC

**FIGURE 11:**
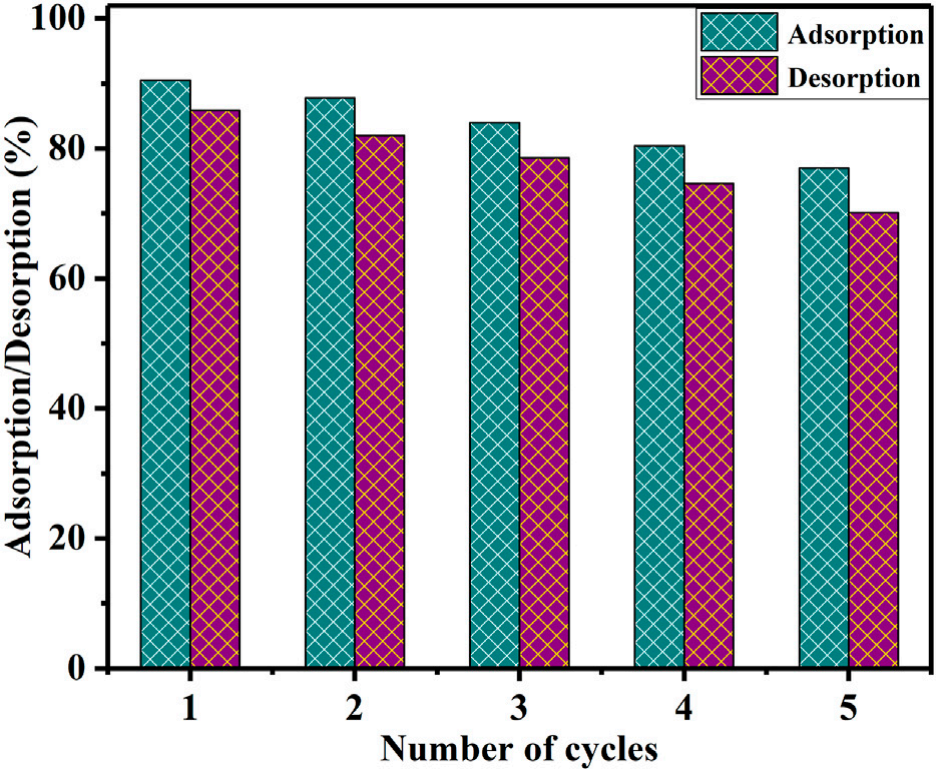
Adsorption-desorption cycles of U(VI) ions onto BAC.

Regenerating used sorbents offers several advantages, including cost-effectiveness, minimized disposal expenses, decreased environmental risks, and the opportunity to recover the absorbed metals (Gedam and Dongre, 2016). The investigation of reusing the BAC adsorbent in the extraction of U(VI) ions is conducted. U(VI) ions adsorbed on BAC were desorbed by initially rinsing with deionized water, followed by washing with 0.1 M HCl. To initiate the desorption process, 1.0 g of U(VI)-loaded BAC was rinsed with distilled water, air-dried, and then placed in contact with a 0.1 M HCl desorption agent in 50 mL flask. To achieve equilibrium, the flasks were agitated at room temperature, maintaining a speed of 200 rpm, for a duration of 2 h. Considering the prior information, the BAC was subjected to five consecutive adsorption and desorption cycles (Yang et al., 2019). As depicted in Figure 11, the percentage of adsorption altered from 90.8% to 77.0%. Meanwhile, the percentage of desorption shifted from approximately 85.9% to 70.13%. These outcomes underscore the robust stability of the utilized BAC over five consecutive adsorption and desorption cycles.

### Extraction mechanism

**FIGURE 12:**
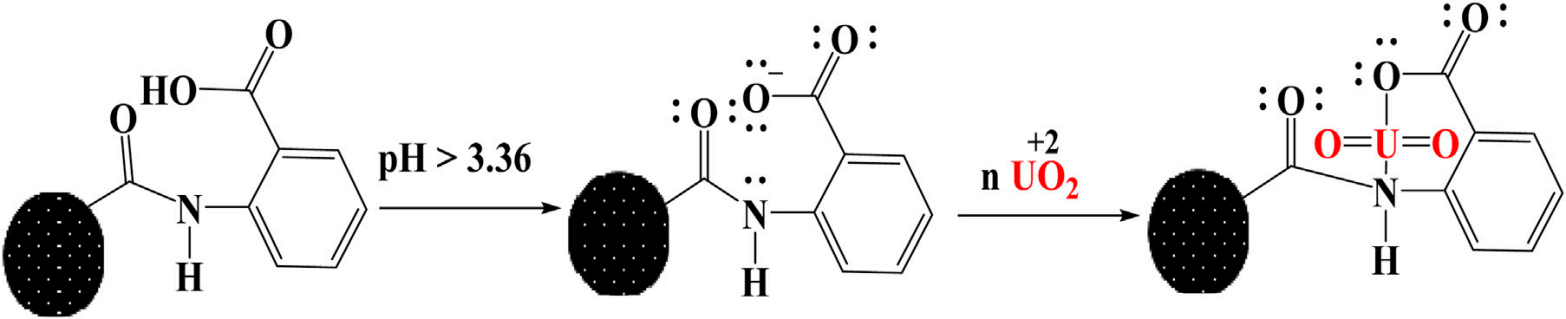
Adsorption interaction mechanism of U(VI) onto BAC.

The considerable impact of pH on extraction ability demonstrates that UO_2_^2+^ absorption onto BAC is controlled by surface functionalities as a result of their protonation or deprotonation activities. At low pH levels (less than pH_PZC_), a low interaction between U (VI) and BAC was found due to significant protonation activity of the -COOH and -OH functional groups, resulting in limited adsorption onto BAC (Cheng et al., 2019). On the other hand, as the pH of BAC exceeds pHPZC (3.36), deprotonation of -COOH groups on the BAC surface takes place, which leads to high interaction of U(VI) with BAC due to the availability of lone pair electrons and the presence of vacant orbitals on uranyl ions, as shown in schematic (Figure 12).

## Conclusion

In this research mesoporous high surface area biowaste activated carbons (BAC) were successfully derived from local biowaste (Albizia Lebbeck Pods) by chemical activation using phosphoric acid. The resulting activated carbon exhibited favorable U(VI) ions extraction behavior from aqueous solutions. The high S_BET_ 1405.95 m^2^/g, t-plot S_Mic._ 128.13 m^2^/g, t-plot S_Meso._ 1277.82 m^2^/g, V_P. Tot._ 1.276 cm^3^/g, t-Plot V_Mic._ 0.048 cm^3^/g and t-Plot V_Meso._ 1.228 cm^3^/g of BAC were achieved at 24 h soaking time, 400°C carbonization temperature and 3 h carbonization time. The BAC’s additional surface features were validated using XRD, FTIR, SEM, and EDX analysis. The study investigated the influence of numerous adsorption factors such as solution pH, contact duration, BAC dosage and concentrations of U(VI). The highest extraction efficiency, reaching 90.60% for U(VI) ions, was achieved using parameters: BAC dosage of 0.5 g, solutions pH 6, a contact time of 120 min and U(VI) ion concentration of 10 ppm at T_R_. The study resulted in the production of cost-effective and ecofriendly BAC. The extraction study has affirmed its efficacy as an adsorbent for U(VI) extraction from aqueous solutions. The BAC adsorbent material demonstrated efficient regeneration and reusability for U(VI) extraction in multiple sorption-desorption cycles, retaining its extraction ability without significant loss. The development of this effective adsorbent derived from biowaste could be promising advancement for uranium adsorption to address the substantial demand for uranium in nuclear energy applications. However, some of the challenges including micropores clogging and post adsorption affinity to uranium should be addressed.

## Data Availability

The original contributions presented in the study are included in the article/supplementary material, further inquiries can be directed to the corresponding author.
